# Dual resistance to carbapenems and colistin in *Enterobacter*: Taiwan surveillance of antimicrobial resistance, 2010–2020

**DOI:** 10.1080/22221751.2026.2623693

**Published:** 2026-02-10

**Authors:** Ying-Chi Huang, Tzu-Wen Huang, Praveen Rahi, Shu-Chen Kuo, Yan-Ru Chen, Chi-Tai Fang

**Affiliations:** a National Institute of Infectious Diseases and Vaccinology, National Health Research Institutes, Zhunan, Taiwan; b Institute of Epidemiology and Preventive Medicine, College of Public Health, National Taiwan University, Taipei, Taiwan; c Department of Microbiology and Immunology, School of Medicine, College of Medicine, Taipei Medical University, Taipei, Taiwan; d Graduate Institute of Medical Sciences, College of Medicine, Taipei Medical University, Taipei, Taiwan; e Bacteriology and Antibiotic Resistance, Medical Biology Laboratory, Institut Pasteur du Cambodge, Phnom Penh, Cambodia; f Division of Infectious Diseases, Department of Internal Medicine, National Taiwan University Hospital, Taipei, Taiwan

**Keywords:** *Enterobacter* spp, colistin resistance, carbapenem resistance, *arn* operon, *mcr-9*, *mcr-10*, IncHI2 plasmid

## Abstract

Extensively drug-resistant gram-negative bacteria harbouring dual resistance to carbapenems and colistin represent a critical global health threat. A total of 929 population-representative *Enterobacter* isolates were systematically collected from 29 hospitals across four regions of Taiwan between 2010 and 2020. Forty-one isolates (4.4%) were nonsusceptible to carbapenems and underwent whole-genome sequencing, resistance gene profiling, plasmid analysis, and antimicrobial susceptibility testing (AST). Among them, 35 isolates (85.4%) exhibited dual resistance to carbapenems and colistin; however, only half (17/35) were detectable by standard phenotypic AST. Colistin resistance was primarily mediated by activation of the chromosomal *arnBCADTEF* operon, which was frequently inducible and often undetected by standard testing, rather than by *mcr-9* or *mcr-10*. A conserved IncHI2 plasmid carrying *bla*_IMP-8_ and *mcr-9* persisted and circulated across *Enterobacter* species for over a decade. Species-specific resistance patterns were observed: *E. roggenkampii* typically exhibited colistin resistance despite lacking carbapenemases, whereas *E. hormaechei* commonly carried *bla*_IMP-8_ and occasionally lacked the *arn* operon. Both species exhibited comparable imipenem nonsusceptibility, complicating therapeutic decision-making. The convergence of carbapenem and colistin resistance in a substantial proportion of *Enterobacter* isolates at the population level makes this genus an emerging priority for hospital infection control and antimicrobial resistance surveillance. These findings underscore the urgent need for improved diagnostics, strengthened antimicrobial resistance surveillance, and optimized treatment strategies.

## Introduction

*Enterobacter* species have emerged as important nosocomial pathogens, ranking third among *Enterobacterales* after *Escherichia coli* and *Klebsiella pneumoniae* [1–3]. *Enterobacter* bacteraemia is associated with attributable mortality rates approaching 40% [4]. Treatment is challenging because intrinsic AmpC *β*-lactamase production and widespread fluoroquinolone resistance limit therapeutic options, leaving carbapenems as essential agents [5]. Alarmingly, carbapenem resistance in *Enterobacter* is increasing worldwide, mediated by carbapenemases or by AmpC/ESBL production combined with porin loss [3,6].

Colistin, often reserved as a last-line agent, is now also facing increasing resistance in *Enterobacter* spp. [7–9]. Plasmid-mediated *mcr* genes (*mcr-1* to *mcr-10*) have emerged as transferable determinants of colistin resistance in *Enterobacterales*, with *mcr-9* and *mcr-10* especially relevant in *Enterobacter* spp. [7,10]. Doijad et al. recently demonstrated that colistin resistance in *Enterobacter* can also be mediated by the chromosomal *arnBCADTEF* operon (also named *pmrHFIJKLM* operon), which modifies lipid A through the addition of 4-amino-4-deoxy-L-arabinose (L-Ara4N), in isolates from German hospitals [9]. Expression of the *arn* operon is regulated mainly by PhoPQ two component system and can be induced upon colistin exposure, resulting in resistant subpopulations escape detection by routine susceptibility testing [8,9]. Both *in vivo* and clinical treatment failure have been documented in association with this mechanism [11,12]. Notably, a high prevalence of colistin heteroresistance (approximately 28%) has been reported among *Enterobacter* clinical isolates in Japan, with clustering in specific species and lineages within a single medical centre [13]. Despite detailed mechanistic characterization, the population-level prevalence and clinical impact of *arn*-mediated colistin resistance remain poorly defined at a global scale.

In Taiwan, the nationwide Taiwan Surveillance of Antimicrobial Resistance (TSAR), established in 1998, has systematically collected clinical isolates from more than 25 hospitals across four geographic regions [2,3]. Carbapenem resistance in *Enterobacter* has been linked to *bla*_IMP-8_, a metallo-*β*-lactamase that compromises most novel β-lactam/β-lactamase inhibitor combinations [3]. The convergence of carbapenemase *bla*_IMP-8_ with colistin resistance would leave few therapeutic options.

This study aimed to describe this emerging antimicrobial resistance pattern of major global concern – the convergence of carbapenem and colistin resistance in *Enterobacter*, using the population-based, multicentre TSAR programme. We further characterized the genetic basis of this dual resistance through whole-genome sequencing.

## Methods

### Isolate collection

Isolates were obtained through the TSAR programme from 29 hospitals across 4 regions of Taiwan between 2010 and 2020 biennially [2,3]. Participating hospitals comprised 12 medical centres and 17 regional hospitals. In each surveillance year, bacterial isolates were collected sequentially from clinical specimens between July and September without species preselection. All isolates were identified by MALDI-TOF mass spectrometry (Bruker Daltonics, USA), and antimicrobial susceptibility testing (AST) was performed following species identification. Only isolates identified as *Enterobacter* spp. were included in the present study. Demographic and clinical information provided by the hospitals included patient age, specimen type, and ward type (intensive care unit [ICU], non-ICU inpatient ward, or outpatient setting including the emergency department). Patients were categorized into three age groups: children (<18 years), adults (18–64 years), and elderly (≥65 years). The protocol was approved by the Institutional Review Board of the National Health Research Institutes (EC1101105-E).

### Antimicrobial susceptibility testing (AST)

Minimal inhibitory concentrations (MIC) to amikacin, aztreonam, cefepime, cefotaxime, ciprofloxacin, colistin, gentamicin, ertapenem, imipenem, meropenem, levofloxacin, piperacillin–tazobactam, tigecycline, and trimethoprim–sulfamethoxazole were determined by broth microdilution (Thermo Fisher Scientific, UK) according to CLSI guidelines [14]. Susceptibility to newer β-lactam/β-lactamase inhibitor combinations (meropenem–vaborbactam, imipenem–relebactam, and ceftazidime–avibactam) was determined by in-house broth microdilution.

The U.S. CDC carbapenem-resistant *Enterobacterales* (CRE) definitions define CRE as exhibiting non-susceptibility to at least one carbapenem or evidence of carbapenemase production [15]. Accordingly, in the present study, isolates with imipenem MIC ≥ 2 µg/mL were classified as carbapenem non-susceptible [14], because some isolates classified as susceptible to meropenem were not susceptible to imipenem. Colistin MICs ≤ 2 µg/mL were interpreted as wild-type susceptibility [14], and tigecycline non-susceptibility was defined as MIC > 2 µg/mL according to EUCAST breakpoints(https://www.eucast.org/clinical_breakpoints).

### Broth macrodilution of colistin susceptibility and inducible resistance testing

Colistin susceptibility testing was performed by the broth macrodilution method in accordance with CLSI guidelines [16]. Sterile PYREX® glass test tubes (12 × 75 mm) fitted with metal closures were used for all dilutions. Bacterial isolates were obtained from overnight cultures on blood agar plates. Three to five well-isolated colonies of identical morphology were selected and suspended in sterile saline. The turbidity of the bacterial suspension was adjusted to a 0.5 McFarland standard. Antimicrobial stock solutions were prepared so that the series of colistin dilutions were at twice the desired final concentrations, to account for the 1:2 dilution resulting from addition of equal volumes of inoculum. The 0.5 McFarland suspension was diluted 1:150 in cation-adjusted Mueller–Hinton broth to yield a suspension of approximately 1 × 10^6^ CFU/mL. One millilitre of this inoculum was then added to each test tube containing 1 mL of the appropriate colistin concentration (and to a control tube containing 1 mL of broth only), resulting in a final inoculum of 5 × 10^5^ CFU/mL. Tubes were incubated at 35 ± 2 °C for 16–20 h, and the MIC was recorded as the lowest concentration of colistin that completely inhibited visible bacterial growth. The skipped-well phenomenon was defined as a non-monotonic growth pattern during broth macrodilution testing, characterized by regrowth at higher colistin concentrations. When this phenomenon was observed, MIC values were assigned as the lowest colistin concentration that inhibited visible bacterial growth without regrowth at higher concentrations.

To further evaluate colistin inducible resistance, LB-colistin agar spot assay was conducted using LB agar supplemented with 2 µg/mL colistin (LA_2 µg/mL) [17]. Overnight LB cultures (pH 7.0) were adjusted to an optical density of OD₆₀₀ = 0.9. A 1:10 dilution was prepared by mixing 100 µL of culture with 900 µL phosphate-buffered saline (PBS). From each of the three sources – (i) overnight culture, (ii) OD₆₀₀ = 0.9 refreshed culture, and (iii) 1:10 dilution – 10 µL aliquots were spotted onto LA_2 µg/mL plates. After air-drying, the plates were incubated at 37 °C for 16–20 hours. The appearance of discrete colonies or hazy growth was interpreted as evidence of colistin-tolerant or heteroresistant subpopulations. All experiments were performed in triplicate to ensure reproducibility.

### Sample preparation, DNA extraction, and whole genome sequencing (WGS)

From 2010 to 2020, a total of 929 *Enterobacter* isolates were collected, of which 41 were imipenem non-susceptible. Single colonies of these 41 isolates were cultured overnight and DNA was extracted using the Promega Wizard® Genomic DNA Purification Kit. WGS was performed on the Pacific Biosciences Sequel II platform (https://www.pacb.com). Genome assembly was conducted using ≥150 Mb of HiFi reads (>5 kb) with the Genome Assembly tool in PacBio SMRTLink version 10.2. The assembled genomic sequences were deposited under BioProject accession number PRJNA874798, with accession numbers for individual isolates provided in Supplementary Table S1. Twelve reference genomes used for comparative analysis are provided in Supplementary Table S2 [18].

### Species identification and phylogenetic analysis

Species assignment was confirmed by *in silico* DNA–DNA hybridization (*is*DDH) using the Genome-to-Genome Distance Calculator (GGDC, cutoff 70%) [19] and by Average Nucleotide Identity (ANI) using QIAGEN CLC Genomics Workbench v9.5.3 [20]. Results are provided in Supplementary Table S1. Core-genome alignment was generated using Panaroo v1.5.2, defining core genes as those present in 99–100% of isolates, yielding 2,760 core genes for phylogenetic reconstruction [21]. Maximum-likelihood phylogenetic analysis was performed using IQ-TREE v2.1.4 with automatic model selection (ModelFinder) based on the concatenated core-gene alignment [22]. Branch support was assessed using 1,000 ultrafast bootstrap replicates and 1,000 SH-aLRT tests (-m MFP – bb 1000 – alrt 1000 – nt AUTO). Support values are shown on the final tree, which was visualized using iTOL v6 webserver (https://itol.embl.de/).

### Genomic characterization and comparative analysis

Genome annotation including insertion sequence, virulence gene and integrase was performed using the NCBI Prokaryotic Genome Annotation Pipeline (PGAP) [23]. Multilocus sequence typing (MLST) was carried out using MLST 2.0 [24]. Comprehensive Antibiotic Resistance Database (CARD) was used to detect acquired antimicrobial resistance genes [25]. Plasmid replicon types were determined using the PubMLST plasmid MLST database (https://pubmlst.org/organisms/plasmid-mlst; accessed on 2025/05/27). Plasmid comparisons were also analysed using BRIG (BLAST Ring Image Generator) to visualize structural similarities and differences among plasmid sequences [26]. Promoter prediction was predicted using BPROM [27]. Nucleotide sequence alignments were performed with BLAST [28], and genomic context visualizations were generated with EasyFig [29].

### Statistical analysis

The Cochran–Armitage trend test was used to evaluate temporal trends in the proportion of isolates classified as imipenem non-susceptible versus imipenem susceptible, with year of surveillance treated as an ordinal variable. Proportions of clinical characteristics, resistance genes, and antimicrobial susceptibility were compared between *E. hormaechei* and *E. roggenkampii* isolates using Fisher’s exact test. Two-sided *P* values <0.05 were considered statistically significant. All statistical analyses were performed using R version 4.3.2 (R Foundation for Statistical Computing, Vienna, Austria).

## Results

### Species identification and epidemiology

**Figure 1.**
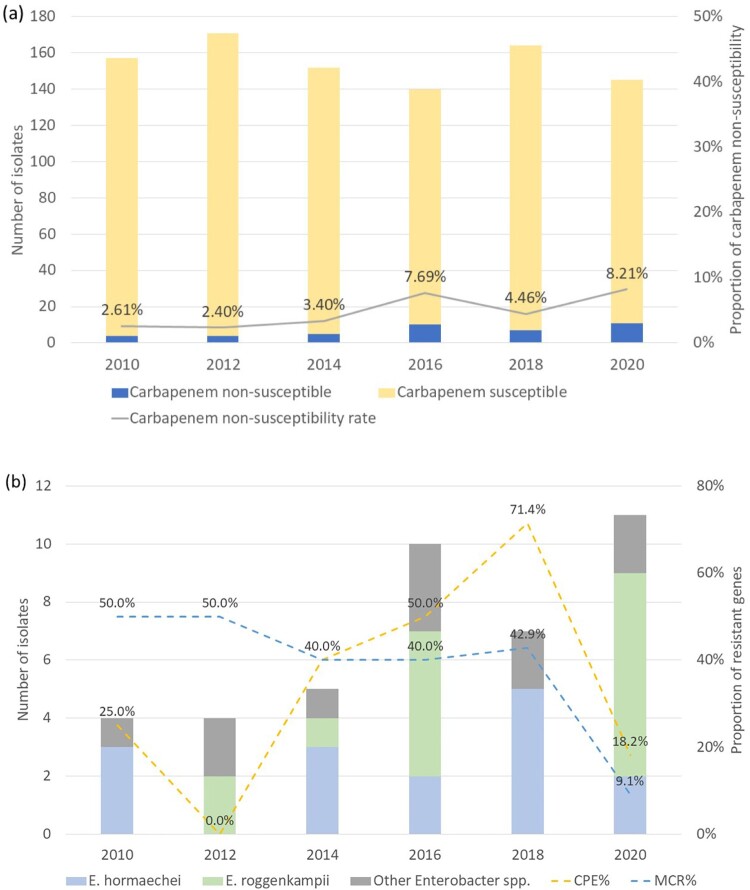
(A) Carbapenem non-susceptibility among 929 *Enterobacter* isolates in Taiwan (2010–2020). Proportion of carbapenem-non-susceptible isolates (grey line). (B) Species distribution of carbapenem non-susceptible *Enterobacter* isolates, mainly *E. roggenkampii* and *E. hormaechei.* The yellow dashed line indicates the proportion of carbapenemase producing isolates. The blue dashed line indicates the proportion of *mcr* gene carriage.

From 2010 to 2020, 929 *Enterobacter* isolates were collected, of which 41 (4.6%) were imipenem non-susceptible (32 intermediate, MIC = 2 µg/mL; 9 resistant, MIC > 2 µg/mL). The proportion of imipenem non-susceptible isolates increased over the study period, peaking at 8.2% in 2020 (Cochran–Armitage trend test, *P* = 0.013) (Figure 1a).

Among the 41 isolates, blood was the most common source (39%), followed by urine (22%), pus (19.5%), and sputum (14.6%). Rare sources included ascites, catheter tips, and unknown origins, with one isolate from each. Nearly half of the isolates were obtained from elderly patients (≥65 years, 48.8%), and most were recovered from inpatients (75.6%).

**Figure 2.**
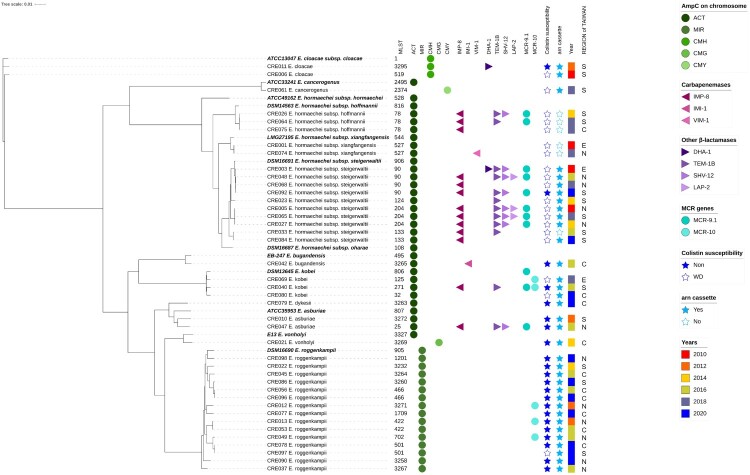
Phylogenetic tree of 41 carbapenem-non-susceptible *Enterobacter* isolates collected in Taiwan (2010–2020). Reference strains are shown in bold, and isolates from this study are in regular font. WD indicates colistin wild type (MIC ≤ 2 µg/mL). Resistance determinants, *arn* operon, *mcr* genes, and year of collection are annotated alongside each isolate.

WGS-based identification revealed equal numbers of *E. hormaechei* and *E. roggenkampii* (15 each, 36.6%), with the remainder including three *E. kobei* (7.3%), two each of *E. asburiae* and *E. cloacae* (4.9% each), and one isolate (2.4%) each of *E. bugandensis, E. cancerogenus, E. vonholyi,* and *E. dykesii* (Figure 1b). Core-genome phylogenetic analysis demonstrated clear species-level clustering, with all clinical isolates forming well-supported monophyletic clades corresponding to their respective reference strains (Figure 2). Consistently, all pairwise ANI comparisons among clinical isolates formed distinct clusters consistent with species-level cutoffs (≥95–96%)**,** supporting robust species delineation (Supplementary Figure S1).

Within the *E. hormaechei* complex (*n* = 15), isolates further segregated into three distinct subspecies clades: subsp. *steigerwaltii* (*n* = 10), subsp. *hoffmannii* (*n* = 3), and subsp. *xiangfangensis* (*n* = 2), each clustering tightly with its corresponding type strain (accession numbers in Supplementary Table S2). These assignments were further supported by high within-subspecies genomic similarity, with ANI values >98.9% and *is*DDH values >90% (Supplementary Table S1).

Phylogenetic analysis showed that isolates were geographically interspersed across Taiwan, with no evidence of regional clustering (Figure 2). Isolates from the same species, subspecies, and sequence types were distributed across multiple regions, and carbapenemase genes were predominantly detected in *E. hormaechei* across diverse sequence types (ST78, ST90, ST133, ST204).

### Antimicrobial susceptibility, resistant genes, and virulence genes

Clinical characteristics, antimicrobial susceptibility profiles, and resistance determinants of *E. hormaechei*, *E. roggenkampii*, and other *Enterobacter* species are summarized in Table 1. No significant differences in clinical characteristics were observed between *E. hormaechei* and *E. roggenkampii* (all *P* > 0.05).

**Table 1.** Comparison of clinical characteristics, antimicrobial susceptibility, and resistance genes between *E. hormaechei*, *E. roggenkampii*, and other *Enterobacter* spp.

	*E. hormaechei* (EH)	*E. roggenkampii* (ER)	Other *Enterobacter* spp.	*P* - value (EH v.s ER)	Total
Number (%)	15 (36.6)	15 (36.6)	11 (26.8)		41 (100)
Year					
2010–2014	6 (40)	3 (20)	4 (36.4)	0.213	13 (31.7)
2016–2020	9 (60)	12 (80)	7 (63.6)		28 (68.3)
Age group				0.655	
Children	2 (13.3)	1 (6.7)	0 (0)		3 (7.3)
Adult	6 (40)	6 (40)	5 (45.5)		17 (41.5)
Elderly	6 (40)	8 (53.3)	6 (54.5)		20 (48.8)
Unknown	1 (6.7)	0 (0)	0 (0)		1 (2.4)
Hospital type				0.5	
Medical centre	8 (53.3)	7 (46.7)	4 (26.7)		19 (46.3)
Regional hospital	7 (46.7)	8 (53.3)	7 (46.7)		22 (53.7)
Region				0.064	
Central	1 (6.7)	6 (40)	4 (36.4)		11 (26.8)
Eastern	2 (13.3)	0 (0)	1 (9.1)		3 (7.3)
Northern	5 (33.3)	6 (40)	1 (9.1)		12 (29.3)
Southern	7 (46.7)	3 (20)	5 (45.5)		15 (36.6)
Source				0.251	
Blood	4 (26.7)	7 (46.7)	5 (45.5)		16 (39)
Pus	2 (13.3)	3 (20)	3 (27.2)		8 (19.5)
Respiratory	3 (20)	3 (20)	0 (0)		6 (14.6)
Urine	6 (40)	1 (6.7)	2 (18.2)		9 (22.0)
Other	1 (6.7)	1 (6.7)	1 (9.1)		3 (7.3)
Invasive	3 (20)	8 (53.3)	5 (45.5)	0.064	16 (39)
Location				0.648	
ICU	4 (26.7)	2 (13.3)	0 (0)		6 (14.6)
Non-ICU inpatient	9 (60)	11 (73.3)	11 (100)		31 (75.6)
Outpatient (OPD and ER)	2 (13.3)	2 (13.3)	0 (0)		4 (9.8)
Antimicrobial non-susceptibility					
Amikacin	3 (20)	0 (0)	0 (0)	0.112	3 (7.3)
Gentamicin	9 (60)	0 (0)	2 (18.2)	<0.001	11 (26.8)
Aztreonam	13 (86.7)	4 (26.7)	3 (27.3)	0.001	20 (48.8)
Cefotaxime	14 (93.3)	4 (26.7)	6 (54.5)	<0.001	24 (58.5)
Cefepime	12 (80)	0 (0)	1 (9.1)	<0.001	13 (31.7)
Ciprofloxacin	12 (80)	5 (33.3)	2 (18.2)	0.013	19 (46.3)
Trimethoprim/sulfamethoxazole	9 (60)	0 (0)	2 (18.2)	<0.001	11 (26.8)
Piperacillin/ tazobactam	14 (93.3)	3 (20)	1 (9.1)	<0.001	18 (43.9)
Tigecycline	10 (66.7)	1 (6.7)	0 (0)	0.001	11 (26.8)
Colistin^a^	0 (0)	12 (80)	5 (45.4)	<0.001	17 (41.5)
Meropenem/ vaborbactam	0 (0)	0 (0)	0 (0)	-	0 (0)
Imipenem/ relebactam	12 (80)	0 (0)	4 (36.4)	<0.001	16 (39)
Ceftazidime/ avibactam	12 (80)	0 (0)	2 (18.2)	<0.001	14 (34.1)
Antimicrobial resistance gene					
ESBL or AmpC gene carried on plasmid	10 (66.7)	0 (0)	2 (18.2)	<0.001	12 (29.3)
Carbapenemase gene^b^	12 (80)	0 (0)	3 (27.3)	<0.001	15 (36.6)
Chromosomal *arn* gene	9 (60)	15 (100)	11 (100)	0.017	35 (85.4)
*mcr* gene^c^	8 (53.3)	3 (20)	3 (27.3)	0.064	14 (34.1)

^a^ Colistin MIC was done by broth microdilution.

^b^ Carbapenemase genes included *bla*_IMP-8_ (*n* = 11) and *bla*_VIM-1_ (*n* = 1) in *E. hormaechei*, *bla*_IMP-8_ in *E. kobei* and *E. asburiae*, and *bla*_IMI-1_ in *E. bugandensis*. No carbapenemase genes were detected in the two *E. cloacae* isolates.

^c^ All three *E. roggenkampii* isolates harboured the *mcr-10* gene, and all eight *E. hormaechei* isolates carried *mcr-9*. Among the remaining *Enterobacter* species, one *E. kobei* co-harboured *mcr-9* and *mcr-10*, another *E. kobei* harboured *mcr-10* only, and one *E. asburiae* harboured *mcr-9* only.

Compared with *E. roggenkampii*, *E. hormaechei* isolates showed significantly higher resistance to aztreonam, cefotaxime, cefepime, ciprofloxacin, trimethoprim–sulfamethoxazole, tigecycline, and piperacillin/tazobactam (all *P* ≤ 0.025; ORs 8.0–120.0). Notably, 80% of *E. hormaechei* isolates were resistant to imipenem/relebactam or ceftazidime/avibactam, whereas none of the *E. roggenkampii* isolates showed resistance to these agents (*P* < 0.001). In contrast, colistin resistance was markedly more prevalent in *E. roggenkampii* than in *E. hormaechei* (80% vs. 0%, *P* < 0.001).

Genotypically, the chromosomal *arn* operon was universally present in all *Enterobacter* isolates except for six *E. hormaechei* isolates. Plasmid-mediated ESBL/AmpC and carbapenemase genes were confined mostly to *E. hormaechei* (80%). Although *mcr* genes were more frequently detected in *E. hormaechei* (53.3% vs 20.0%), this difference did not reach statistical significance (*P* = 0.128). The overall *mcr* carriage rate across *Enterobacter* isolates declined from 40–50% before 2018 to 9.1% by 2020 (Figure 1b).

Analysis of virulence-associated genes revealed that most isolates carried conserved adhesion – and biofilm-related determinants, including *fimH*, *ompA*, *ompC*, and the curli operon (*csgA/csgG/csgD*) (Supplementary Table S1).

### Chromosomal mechanisms of colistin resistance

Colistin MICs showed no consistent correlation with *mcr-9*/*mcr-10* carriage but were strongly associated with the chromosomal a*rnBCADTEF* operon (Table 1). Among the 41 isolates, 35 (85.4%) carried the *arn* operon, while six *E. hormaechei* lacked it (Supplementary Table S1). All *arn*-negative strains remained susceptible (MIC ≤ 0.25–1 µg/mL), whereas *arn*-positive isolates exhibited variable MICs ranging from ≤0.25 to >4 µg/mL. All isolates retained intact *mgrB* and *phoPQ* genes without truncations, indicating these were not primary drivers of resistance.

Table 2 and Supplementary Figure S2 summarize colistin susceptibility testing results and inducible resistance phenotypes among representative isolates with different *arn* operon statuses and genetic backgrounds. Isolates lacking the *arnBCADTEF* operon, including *E. hormaechei* subsp. *xiangfangensis* (ST527) and subsp. *hoffmannii* (ST78), consistently exhibited low colistin MICs (≤0.125–0.25 µg/mL), showed no skipped-well phenomena, and failed to grow on colistin-containing agar (2 µg/mL), indicating stable susceptibility. In contrast, multiple *arn*-positive isolates, particularly *E. hormaechei* subsp. *steigerwaltii* ST90 and *E. roggenkampii* ST501, demonstrated discordant susceptibility profiles, characteristic skipped-well phenomena in broth macrodilution assays and growth on colistin-containing agar, consistent with inducible colistin resistance.

**Table 2.** Colistin susceptibility and inducible resistance among *Enterobacter* spp. (extracted from Supplementary Table S1 and Fig S3).

Isolate	*Enterobacter* species	MLST	Colistin MIC range (µg/mL)^a^	*mcr* genes	*arn* operon	Promoter of *arn* operon^b^	Skip well	Colistin MHA 2 µg/mL	Interpretation
CRE097	*E. roggenkampii*	ST501	0.25∼> 4	−	+	Type 1	+	Colony growth	Inducible resistance
CRE078	*E. roggenkampii*	ST501	> 4	−	+	Type 1	−	Colony growth	Persistent resistance
CRE001	*E. hormaechei* subsp. *xiangfangensis*	ST527	≤ 0.125	−	−	NA	−	−	No resistance
CRE074	*E. hormaechei* subsp. *xiangfangensis*	ST527	≤ 0.125∼0.25	−	−	NA	−	−	No resistance
CRE003	*E. hormaechei* subsp. *steigerwaltii*	ST90	0.125∼1	+	+	Type 2	−	Colony growth	Inducible resistance
CRE092	*E. hormaechei* subsp. *steigerwaltii*	ST90	0.125∼4	+	+	Type 2	+	Colony growth	Inducible resistance
CRE048	*E. hormaechei* subsp. *steigerwaltii*	ST90	≤ 0.125∼2	+	+	Type 2	+	Colony growth	Inducible resistance
CRE068	*E. hormaechei* subsp. *steigerwaltii*	ST90	≤ 0.125	−	+	Type 2	+	Colony growth	Inducible resistance
CRE026	*E. hormaechei* subsp. *hoffmannii*	ST78	≤ 0.125∼0.25	+	−	NA	−	−	No resistance
CRE064	*E. hormaechei* subsp. *hoffmannii*	ST78	≤ 0.125	+	−	NA	−	−	No resistance
CRE075	*E. hormaechei* subsp. *hoffmannii*	ST78	≤ 0.125	−	−	NA	−	−	No resistance
CRE033	*E. hormaechei* subsp. *steigerwaltii*	ST133	≤ 0.125	−	−	NA	−	−	No resistance
CRE084	*E. hormaechei* subsp. *steigerwaltii*	ST133	≤ 0.125	−	+	Other	−	−	No resistance

+, present; −, absent; NA, not applicable.

^a^ The MIC range was determined in triplicate using the broth macrodilution method.

^b^ Type 1 promoter: attggtttaaatgtttccatttcaaaatgttgcggaagatcacatc; Type 2 promoter: tttattggaaatggtcggggatttttttatttgttga; Other promoter details are described in Supplementary Table S1.

Promoter type distribution differed by species. Type 1 promoters, predominant in *E. roggenkampii*, were associated with constitutive or inducible resistance, whereas type 2 promoters, common among *E. hormaechei* ST90 isolates, were linked specifically to inducible resistance. One exceptional *E. hormaechei* isolate (CRE084) harboured a divergent promoter variant, likely explaining the absence of inducible resistance despite an intact *arn* operon. Overall, colistin resistance in these *Enterobacter* isolates was primarily determined by chromosomal *arn* operon regulation with species differences.

### Frequent co-carriage of *bla*_IMP-8_ and *mcr-9* on IncHI2 plasmids

Co-carriage of *bla*_IMP-8_ and *mcr-9* on IncHI2 plasmids persisted for 10 years in this study, representing a long-term stable multidrug resistance platform (Table 3). Overall, 36.6% (15/41) of *Enterobacter* isolates produced carbapenemases, the vast majority carrying *bla*_IMP-8_ (*n* = 13), primarily in *E. hormaechei* (*n* = 11), and in one isolate each of *E. kobei* and *E. asburiae.* Other carbapenemases included *bla*_IMI-1_, identified in a single isolate of *E. bugandensis*, and *bla*_VIM-1_, detected in one isolate of *E. hormaechei*. These IncHI2 plasmids were frequently co-localized with *mcr-9.1* (7/8, 87.5%) and additional β-lactamases such as *bla*_SHV-12_ and *bla*_TEM-1_ (Table 3). *E. hormaechei* ST204 isolates exemplified this archetypal superplasmid, consistently carrying *bla*_IMP-8_, *mcr-9.1*, and additional β-lactamases on the same IncHI2 plasmid. BRIG analysis confirmed a highly conserved IncHI2 ST1 backbone across isolates from different years and regions (2010–2020) (Supplementary Figure S3A). Despite the frequent presence of *mcr* genes, most *bla*_IMP-8_–positive isolates remained colistin-susceptible.

**Table 3.** Characteristics of *bla*_IMP-8_-carrying *Enterobacter* spp.

Species	Isolate	Year	Region	MLST	Sizes of plasmids or inserts (bp)	Location of *bla*_IMP-8_ genes	Co-harbouring ß-lactamase	*mcr* gene	*arn* cassette
*E. hormaechei s*ubsp. *steigerwaltii*	CRE005	2010	N	ST204	326857	IncHI2 ST1	SHV-12, TEM-1	*mcr-9.1*	+
*E. hormaechei s*ubsp. *steigerwaltii*	CRE027	2014	N	ST204	318009	IncHI2 ST1	SHV-12, TEM-1	*mcr-9.1*	+
*E. hormaechei s*ubsp. *steigerwaltii*	CRE065	2018	S	ST204	313832	IncHI2 ST1	SHV-12, TEM-1	*mcr-9.1*	+
*E. hormaechei s*ubsp. *steigerwaltii*	CRE033	2016	S	ST133	161895	FIIY	TEM-1	NA	−
*E. hormaechei s*ubsp. *steigerwaltii*	CRE084	2020	S	ST133	143296	FIIY	NA	NA	+
*E. hormaechei s*ubsp. *steigerwaltii*	CRE048	2016	N	ST90	80734	IncHI2 ST1	NA	*mcr-9.1*	+
*E. hormaechei s*ubsp. *steigerwaltii*	CRE068	2018	N	ST90	154455	FIIY	NA	NA	+
*E. hormaechei s*ubsp. *steigerwaltii*	CRE092	2020	S	ST90	187175	FIIY^a^	TEM-1	*mcr-9.1*^a^	+
*E. hormaechei* subsp. *hofmannii*	CRE026	2014	S	ST78	283759	IncHI2 ST1	SHV-12, TEM-1	*mcr-9.1*	−
*E. hormaechei* subsp. *hofmannii*	CRE064	2018	S	ST78	258855	IncHI2 ST1	TEM-1	*mcr-9.1*	−
*E. hormaechei* subsp. *hofmannii*	CRE075	2018	C	ST78	80458	No match	NA	NA	−
*E. asburiae*	CRE047	2016	N	ST271	278171	IncHI2 ST1	NA	*mcr-9.1*	+
*E. kobei*	CRE040	2016	S	ST25	299622	IncHI2 ST1	TEM-1	*mcr-9.1, mcr-10* ^b^	+

+, present; –, absent; NA, not applicable.

^a^ In this isolate, *mcr-9.1* and *bla*_IMP-8_ were located on different plasmids: *mcr-9.1* on IncHI2 and *bla*_IMP-8_ on IncFIIY plasmid.

^b^ In this isolate, *bla*_IMP-8_ and *mcr-9.1* co-localized on the same IncHI2 plasmid, whereas *mcr-10* was carried on a separate plasmid.

In contrast, IncFIIY plasmids (4/13) carrying *bla*_IMP-8_ rarely co-localized with *mcr* genes, forming a distinct genomic profile. These IncFIIY plasmids showed greater variability in resistance loci and mobile genetic elements (Supplementary Figure S3B).

### Structural conservation of the *bla*_IMP_–*mcr-9* superplasmid

**Figure 3.**
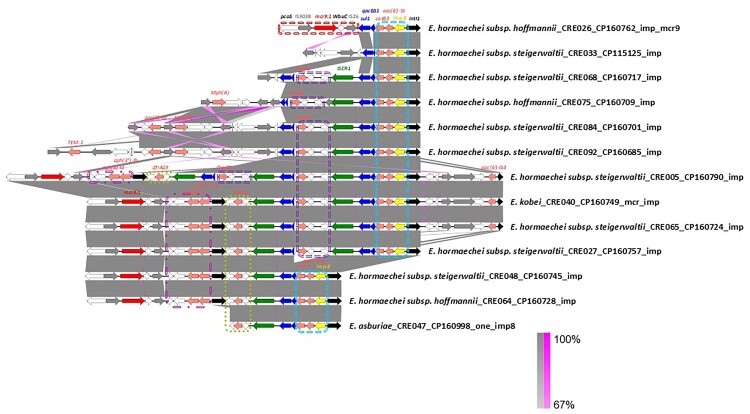
Comparative genomic analysis of the genetic environments of *mcr-9* and *bla*_IMP-8_ among Taiwan *Enterobacter* isolates, 2010–2020. Each horizontal block represents a genomic region containing either the *mcr* or *bla*_IMP_ gene. Arrows indicate open reading frames coloured by functional annotation. Grey shading between sequences indicates regions of shared homology among different plasmids ranging from 80% to 100%. Red: *mcr-9* gene and surrounding genes; Yellow: *bla*_IMP-8_ gene and associated integron elements.

Comparative genomic analysis revealed that all *bla*_IMP-8_ was consistently embedded within a highly conserved class 1 integron (*intI1*–*bla*_IMP-8_–*aac(6”)-Ib*–*catB3*–*qacEΔ1*–*sul1*± IS*CR1*), irrespective of whether the gene resided on IncHI2 or IncFIIY plasmids (Figure 3). This integron was characterized by the presence of the *intI1* integrase at the 5’ conserved segment (5’ CS), followed by a resistance gene cassette comprising *bla*_IMP-8_–aac(6’)-*Ib*–*catB3*, and ending with a 3’ conserved segment (3’CS) containing *qacEΔ1*–*sul1*. In most cases, the cassette was followed by an Insertion Sequence Common Region 1 (IS*CR1*) element, forming a typical complex class 1 integron frequently associated with multidrug resistance. This integron therefore represents the fundamental transmissible unit, while plasmid backbones provided distinct dissemination platforms.

### Global dissemination of *bla*_IMP_–*mcr*-9 superplasmids

**Figure 4.**
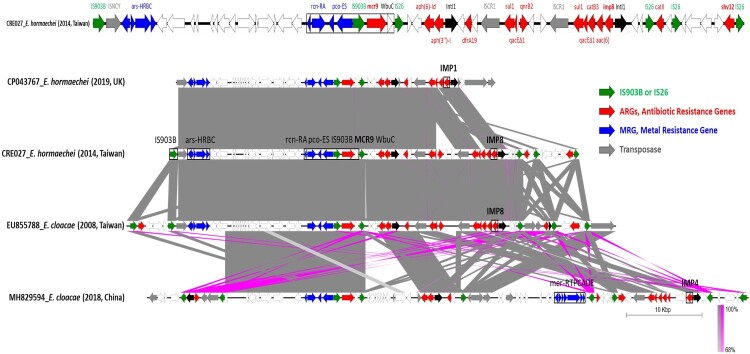
Comparative genomic analysis of global IncHI2 plasmids co-harbouring *mcr-9* and carbapenemase genes in *Enterobacter* spp. Linear representations of plasmid regions are shown with open reading frames (ORFs) depicted as arrows, colour-coded by predicted function: red indicates antibiotic resistance genes (ARGs), purple represents heavy metal resistance genes (MRGs), blue denotes insertion sequences (IS*26*), green for IS*903B e*lements, black/grey for transposases and hypothetical proteins, and uncoloured/white for other coding sequences. Homologous regions with ≥90% nucleotide identity was shaded in grey, with pink connectors indicating shared regions across plasmids.

To place the Taiwan findings in a broader context, we compared these IncHI2 superplasmids with international isolates, demonstrating a globally conserved *bla*_IMP_–*mcr-9* resistance module (Figure 4 and Supplementary Table S3). Across Taiwan, the UK (CP043767), and China (MH829594, MH399264), the *mcr-9* locus was consistently embedded within the IS*903B*–*mcr-9*–*wbuC*–*pco*–*rcn* cluster, linking *mcr-9* gene with copper and arsenic resistance determinants and suggesting co-selection by environmental pressures. This *mcr-9* module was frequently linked to a conserved class 1 integron carrying *bla*_IMP_ and together they formed a composite multidrug resistance region of ∼44–60 kb bracketed by IS26 elements.

### Detection of *mcr-10* and Associated Plasmid Structures

Five isolates (12.2%) carried *mcr-10*, including *E. roggenkampii* (*n* = 3) and *E. kobei* (*n* = 2). As with *mcr-9, mcr-10* carriage did not associate with elevated colistin MICs in our collection. All *mcr-10* genes were located on IncF plasmids, which exhibited heterogeneous architectures with no closely related international counterparts in our comparisons (data not shown).

## Discussion

This nationwide surveillance study revealed that, colistin resistance among carbapenem-non-susceptible *Enterobacter* isolates in Taiwan was primarily mediated by the chromosomal *arn* operon, which was present in 85.4% of isolates. In contrast, carriage of *mcr* genes had little effect on the resistance phenotype. A particularly concerning finding was inducible colistin resistance mediated by the *arn* operon, a phenotype easily missed by routine antimicrobial susceptibility testing. Together, these findings challenge the long-standing assumption that *Enterobacter* species are uniformly susceptible to colistin and underscore the need to re-evaluate the role of colistin in the treatment of infections caused by carbapenem-non-susceptible *Enterobacter*.

From a clinical microbiology perspective, the predominance of chromosomally mediated, inducible colistin resistance has profound implications for routine antimicrobial susceptibility testing. In *Enterobacter*, exposure to colistin can readily activate the chromosomal *arn* operon, resulting in heterogeneous resistance phenotypes that are difficult to detect and that substantially increase the risk of inappropriate therapy. Although CLSI currently categorizes *Enterobacter* species as intrinsically susceptible to colistin [14], resistance rates of 4–20% have been reported in multiple countries [30], suggesting systematic underestimation of true resistance. Taken together, these findings indicate that *Enterobacter* species may be more appropriately regarded as functionally intrinsically resistant to colistin in clinical practice. Accordingly, colistin may be suboptimal for the treatment of *Enterobacter* infections and should be used with caution even when i*n vitro* susceptibility is reported, as exposure may rapidly select for resistance and increase the risk of therapeutic failure [11,12]. Awareness of chromosomal resistance mechanisms and genomic context is therefore essential to guide appropriate antimicrobial decision-making.

Our species-level analysis demonstrated marked heterogeneity in colistin resistance among *Enterobacter* species, consistent with previous *hsp60*-based studies [9,31]. The *arn* operon was universally present in non–*E. hormaechei* species but absent in *E. hormaechei hsp60* clusters III (subsp. *hoffmannii*) and VI (subsp. *xiangfangensis*) [9]. In contrast, *arn* carriage within *E. hormaechei* cluster VIII (subsp. *steigerwaltii*) was heterogeneous, occurring even among isolates sharing the same sequence type (ST133) in the present study. This finding contrasts with a German study reporting uniform *arn* absence in ST133 and suggests regional variation in the genetic composition of these strains [9].

The long-term circulation of a conserved IncHI2 “superplasmid” carrying *bla*_IMP-8_ and *mcr-9* from 2010 to 2020 in Taiwan indicates its stability and dissemination, both clonally within *E. hormaechei* ST204 and ST78 and across other *Enterobacter* species, including *E. asburiae* and *E. kobei*. Similar IncHI2 plasmids co-harbouring *mcr-9* and carbapenemases like *bla*_IMP_ or *bla*_NDM_ have been reported globally [32–34]. The IncHI2 superplasmid has also been identified in other *Enterobacterales*, such as *K. pneumoniae*, *E. coli*, and *Salmonella* [35,36], and is known to be transferable via conjugation [35,37]. Importantly, our findings suggest that complex integrons, particularly those mediated by IS*CR1*, rather than the plasmid backbones themselves, constitute the fundamental transferable units sustaining the dissemination of *bla*_IMP-8_ [38]. Although *bla*_NDM_ was not detected among *Enterobacter* isolates during the study period, NDM-producing *Enterobacter* have been reported in India and China [34,37]. Notably, a recent outbreak reported in 2025 documented the emergence of *bla*_NDM_-harbouring *Enterobacter* in a southern Taiwan hospital, indicating that *bla*_NDM_ has now entered Taiwan and underscoring the need for continued genomic surveillance [39].

This study marks the detection of *mcr-10* in *Enterobacter* species in Taiwan. Unlike *mcr-9*, *mcr-10* was located on IncFIB/FII plasmids and not associated with carbapenemase genes. This finding is consistent with a report from China [40]. Although *mcr-10* did not confer phenotypic resistance on its own, its presence in various animal and environmental reservoirs highlights the importance of a One Health framework that integrates human, animal, and environmental surveillance [41,42]. This illustrates how novel resistance genes can persist and gain significance under selective pressures, even without an immediate clinical impact.

Despite being the most comprehensive genomic dataset of carbapenem-non-susceptible *Enterobacter* in Taiwan to date, this study has limitations. Only 41 isolates were sequenced, which likely underrepresents the true circulating diversity. The lack of functional validation experiments prevents definitive genotype-phenotype confirmation, and clinical outcome data were unavailable. Furthermore, while population analysis profiling (PAP) is the gold standard for heteroresistance, our assays served only as a screening tool and may have underestimated resistant subpopulations.

In conclusion, the convergence of carbapenem non-susceptibility and colistin resistance renders *Enterobacter* a high-risk multidrug-resistant organism. This threat is further compounded by inducible colistin resistance – often undetectable by standard antimicrobial susceptibility testing – and by the long-term persistence of multidrug-resistant plasmids. These findings underscore the urgent need to re-evaluate current therapeutic strategies and to strengthen measures against the spread of these difficult-to-treat resistance mechanisms.

## Supplementary Material

0113 Supplement.xlsx

## Data Availability

Genomic sequences of all 41 isolates have been deposited in the National Center for Biotechnology Information (NCBI) database and their accession numbers are shown in Supplementary Table S1.
